# Fully Automated Quantification of the Striatal Uptake Ratio of [^99m^Tc]-TRODAT with SPECT Imaging: Evaluation of the Diagnostic Performance in Parkinson's Disease and the Temporal Regression of Striatal Tracer Uptake

**DOI:** 10.1155/2015/461625

**Published:** 2015-08-20

**Authors:** Yu-Hua Dean Fang, Shao-Chieh Chiu, Chin-Song Lu, Tzu-Chen Yen, Yi-Hsin Weng

**Affiliations:** ^1^Department of Biomedical Engineering, National Cheng Kung University, Tainan 701, Taiwan; ^2^Center for Advanced Molecular Imaging and Translation, Chang Gung Memorial Hospital, Linkou 333, Taiwan; ^3^Department of Neurology, Chang Gung Memorial Hospital, Linkou 333, Taiwan; ^4^Department of Nuclear Medicine, Chang Gung Memorial Hospital, Linkou 333, Taiwan

## Abstract

*Purpose*. We aimed at improving the existing methods for the fully automatic quantification of striatal uptake of [^99m^Tc]-TRODAT with SPECT imaging.* Procedures*. A normal [^99m^Tc]-TRODAT template was first formed based on 28 healthy controls. Images from PD patients (*n* = 365) and nPD subjects (28 healthy controls and 33 essential tremor patients) were spatially normalized to the normal template. We performed an inverse transform on the predefined striatal and reference volumes of interest (VOIs) and applied the transformed VOIs to the original image data to calculate the striatal-to-reference ratio (SRR). The diagnostic performance of the SRR was determined through receiver operating characteristic (ROC) analysis.* Results*. The SRR measured with our new and automatic method demonstrated excellent diagnostic performance with 92% sensitivity, 90% specificity, 92% accuracy, and an area under the curve (AUC) of 0.94. For the evaluation of the mean SRR and the clinical duration, a quadratic function fit the data with *R* ^2^ = 0.84.* Conclusions*. We developed and validated a fully automatic method for the quantification of the SRR in a large study sample. This method has an excellent diagnostic performance and exhibits a strong correlation between the mean SRR and the clinical duration in PD patients.

## 1. Introduction

Parkinson's disease (PD) is a neurodegenerative disease that results from the loss of dopaminergic neurons in the substantia nigra. It has become a serious health issue that affects 1–1.5% of the elderly population worldwide [1, 2]. Among the available diagnostic tools, neuroimaging is a common procedure for the early diagnosis and further management of PD patients. Common imaging methods used to visualize brain abnormalities in PD include radionuclide imaging [3–6] and magnetic resonance imaging (MRI) [7, 8]. With the use of tracers that specifically bind to the dopamine transporter (DAT) or dopamine receptors, radionuclide imaging evaluates the integrity of the dopamine system in a patient and helps physicians rule out the possibility of other diseases with symptoms similar to PD [3, 5, 9]. For radionuclide imaging, single-photon emission computed tomography (SPECT) has been commonly used in the diagnosis and management of PD patients. In brain scans of PD, common SPECT tracers include ^123^I-*β*-CIT [10, 11], ^123^I-FPCIT [12, 13], ^123^I-Ioflupane (DATSCAN) [6, 14, 15], and [^99m^Tc]-TRODAT [16–18] for DAT imaging and ^123^I-S(-)IBZM [19, 20] for D2 receptors.

Although the visual interpretation of SPECT-based brain scans is the major approach used to interpret findings, quantitative analysis of the images is helpful to determine the diagnosis by providing an objective source of information independent of the image readers. For SPECT scans related to the dopamine system, quantitative analysis can be used to measure tracer uptake in the striatal area [16, 19–26], the size of the properly functional area [27], or the combination of these factors as the “uptake-size index” [28]. Among these indices, the amount or concentration of tracer uptake in the striatal area is the most commonly adapted parameter because it reflects the activity of dopamine transporters or receptors that have been reported as reliable biomarkers for the PD diagnosis. Because of the difficulty in quantifying the radioactivity concentration with SPECT, the striatal uptake concentration is often expressed as a ratio of the mean striatal intensity versus the mean intensity in a reference region, such as the cerebellum, the occipital cortex, or the cerebral cortex. In this report, we use the nomenclature “striatal-to-reference ratio” (SRR) to denote the quantified striatal uptake. Physiologically, the SRR is equivalent to another common term, the “specific uptake ratio” (SUR), which is equal to the SRR minus one. Although several methods and software packages used to quantify the SRR or SUR have been published in the previous decade, the search continues for a clinically appealing algorithm that is fully automated and well validated in a large population and that could be easily implemented or provided as free software [24, 29].

In this work, we present our efforts to develop a novel quantification method for computing the SRR in SPECT images. The aim was to develop an algorithm solution that does not require user intervention while providing reliable tracer uptake quantification through which high diagnostic specificity, sensitivity, and accuracy can be verified in a large study population. The diagnostic performance of our method was evaluated with image data from human subjects. Those image data were also used to evaluate the regression rate of the quantified SRR as a function of PD onset duration.

## 2. Materials and Methods

### 2.1. Study Population

We have retrospectively collected patient data from 426 subjects in this study. The demographical data for all subjects are summarized in Table 1. In brief, the subjects were divided into two groups, PD and nPD. The patients in the PD group fulfilled the “UK Parkinson's Disease Society Brain Bank Clinical Diagnostic Criteria” as “possible” or “probable” PD [30]. For all PD patients, the age at which the PD-related symptoms appeared was obtained through the clinical records and the history provided by the patients. In this study, the clinical duration of PD-related symptoms was defined as the patient age when he or she completed the TRODAT scan subtracted by the age at the onset of PD-related symptoms. The 365 patients in the PD group were further divided into subgroups based on the clinical duration of PD, which included four groups defined by cutoff values of two, five, and ten years. The subgrouping was intended to evaluate the diagnostic performance of the quantified [^99m^Tc]-TRODAT SRR for the discrimination of the PD patients from the nPD group when the clinical duration of the PD patients was considered.

The nPD group consisted of subjects whose dopaminergic systems had remained functionally healthy. Sixty-one subjects were included in this group. Twenty-eight subjects were healthy controls who had been previously recruited for early-phase clinical trials at Chang Gung Memorial Hospital (CGMH), Linkou. Thirty-three subjects were patients with essential tremor (ET). All ET patients were followed for a minimum of one year, and potential PD had been ruled out based on evaluation by an experienced neurologist. A mixture of postural and kinetic tremors was the only clinical symptom in this group of patients who lacked other neurological abnormalities. This retrospective study was approved by the Institutional Review Board of Chang Gung Memorial Hospital (CGMH), Taiwan.

### 2.2. Image Acquisition of [^99m^Tc]-TRODAT SPECT

The [^99m^Tc]-TRODAT was prepared and provided by the Institute of Nuclear Energy Research of Taiwan. All TRODAT scans used in this study were performed in the Department of Nuclear Medicine, CGMH, Linkou. For each subject, 925 MBq [^99m^Tc]-TRODAT was administered intravenously. Four hours after tracer injection, a 50 min scan was performed with a Siemens MULTISPECT or a Siemens ECAM camera. The images were reconstructed using filtered back projection with a ramp-Butterworth filter, a cutoff of 0.3 (cycles/pixel), and an order of 10 using the built-in* syngo* software. Attenuation correction was performed with the conventional Chang method [31]. The pixel size was 2.9 mm in both transverse and axial directions.

### 2.3. SPM-Based SRR Quantification

We initiated the development of our method using spatial normalization within statistical parametric mapping (SPM). As several previous reports have stated, fully automated quantification of SPECT images can be achieved by spatially normalizing the images to a predefined image template in stereotactic coordinates and then calculating the mean intensities with predefined volumes of interest (VOIs) over the image template. This methodology has been shown to provide satisfactory diagnostic results in SPECT [19, 25] and PET [16] with relatively small populations. In addition to evaluating the diagnostic performance with a large population in our study, we also aimed to further improve the SPM-based methods. Rather than applying predefined VOIs to the spatially normalized images to calculate the mean intensity, we utilized the transformation matrices generated during spatial normalization to inversely transform predefined VOIs to the image domain of the original images. The transformed VOIs, which were subsequently aligned with the original data and were applied to the original data to compute the mean intensity. Such mean intensities were then used to calculate the SRR.

### 2.4. Creation of the Normal Template and Template VOIs

**Figure 1:**
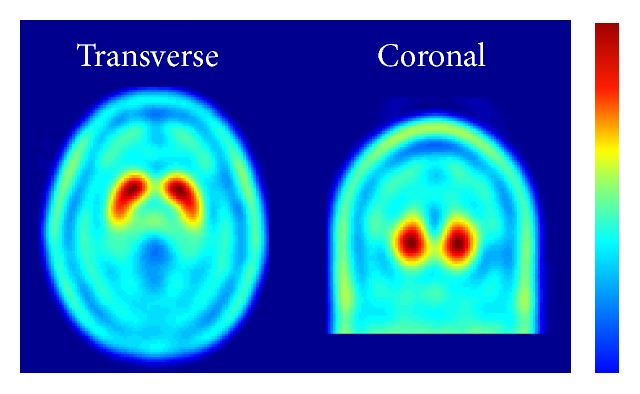
Representative transverse and coronal slices of the [^99m^Tc]-TRODAT template image that was computed from healthy controls.

The TRODAT images obtained from the healthy controls (*n* = 28) were used to form the normal template that was subsequently used as the reference image in the stereotactic coordinates. The normal template was created through the following steps. First, because TRODAT images typically show a nonnegligible uptake in the scalp, the perfusion SPECT template in SPM was modified by adding the scalp segmented from the MRI T1-weighted template that was also included in SPM. Second, with the modified perfusion SPECT as the template image, all TRODAT images obtained from the normal controls were spatially normalized to the same stereotactic coordinates (MNI space). Using SPM spatial normalization procedures previously described [32], we utilized the following parameters for spatial normalization: sixteen iterations, regularization equal to one, 8-mm FWHM for smoothing the source image, and nonsmoothing for the reference image. SPM8 was used in MATLAB R2014a (MathWorks Inc., Natick, MA, USA). Finally, the spatially normalized TRODAT images obtained from the healthy controls were averaged to form the normal template, as shown in Figure 1.

After the normal template was created from the healthy controls, a striatal VOI and reference VOI were generated over the template image. The striatal VOI was created by first masking the template image with a threshold of 60% of the maximum intensity in the template image. The contour of the masked striatum was determined and used to define the striatal VOI on either side. Because the spatial resolution of SPECT is typically insufficient to distinguish the caudate nucleus and putamen, our striatal VOI included both the caudate and putamen without separating them. The cerebral cortex was selected as the reference VOI in this study. Cerebral cortical structures were delineated from the template image with Automated Anatomical Labeling [33] in a selected range of transverse slices. The contour of the segmented cerebral cortex was used to define the VOI of the reference region.

### 2.5. Calculation of the SRR

**Figure 2:**
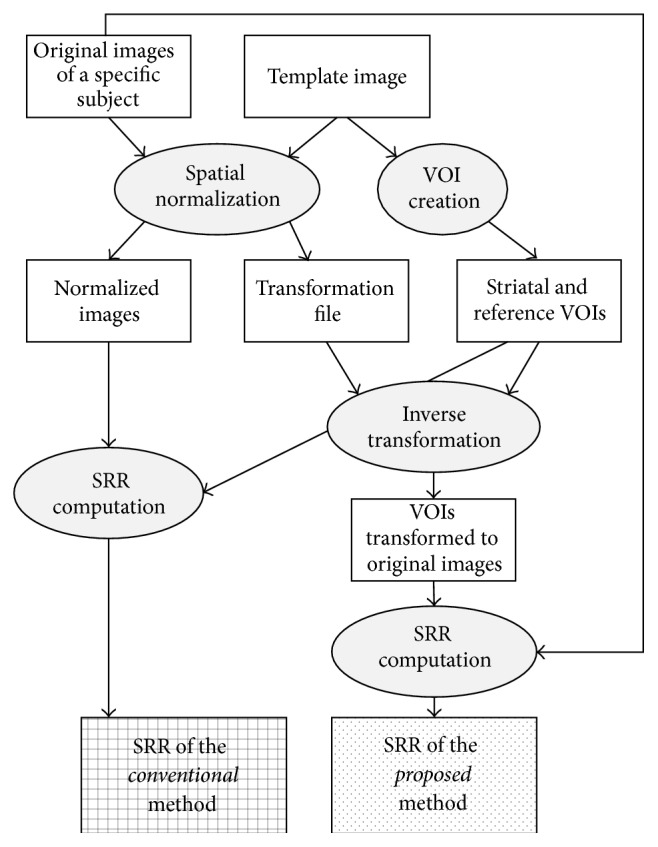
The comparison of the proposed and conventional procedures for computing SRR based on SPM. Data are presented as squares while operations are presented in ovals. In the conventional method, the predefined VOIs are applied on the spatially normalized images. On the other hand, the proposed method performs an inverse transformation of the predefined VOIs to be applied on the original images for SRR calculation.

The SRR was calculated for each subject in the PD and nPD groups. The proposed procedure of the SRR calculation is depicted as a diagram in Figure 2. First, the TRODAT image volume of a specific subject was spatially normalized to the normal template that was created from the healthy controls, as previously described. After spatial normalization, a transformation file was obtained and stored. This transformation file stored multiple transformation matrices that, when multiplied with the images, spatially normalized these images to the standard template domain. Second, the transformation file that resulted from the spatial normalization was used to inversely transform the striatal and reference VOIs from the template image domain to the original image domain. This entailed the individual matrix inversion for all the stored transformation matrices [34], followed by the multiplication of individual inverted matrices to the striatal and reference VOI volumes. Third, once the striatal and reference VOIs were transformed back to the domain of the original image, the mean intensities were calculated from these inversely transformed VOIs. The SRR was then determined by the ratio of the mean striatal intensity divided by the mean reference intensity. In the PD group, the SRR from the contralateral striatum was calculated based on the symptomatic side of a patient. If symptoms were present on both sides for an individual subject, the SRR was calculated from the entire striatum on both sides.

To understand whether our approach provides better diagnostic performance, we also calculated the SRR from spatially normalized images with VOIs defined on the template domain, as described in previous reports [19, 25]. Figure 2 also illustrates the procedure of the conventional procedure.

### 2.6. Determination of the Diagnostic Performance of the SRR

After the SRR was calculated, we used the ROC analysis to determine how accurately the SRR discriminated the PD patients from the nPD subjects. First, the ROC analysis was performed to test the discriminative power of the SRR to separate all PD patients (*n* = 365) from all nPD (*n* = 61) subjects. The area under the curve (AUC) of the ROC curve, the optimal cutoff, and the corresponding sensitivity, specificity, and accuracy were calculated. McNemar's *χ* ^2^ test was used to test whether there is a significant difference in the sensitivity and specificity between our method and the conventional SPM-based method [35]. Second, the individual subgroups of PD subjects (including Group_≤2_, Group_3–5_, Group_6–10_, and Group_>10_) were tested against the nPD subjects in the ROC analysis. Finally, the PD group and its subgroups were tested against the healthy control group. The same analysis was then repeated by testing the PD group and its subgroups against the ET group. The AUC and optimal sensitivity/specificity/accuracy were obtained from the ROC analysis. The mean and standard deviation (SD) of the SRR were also calculated in all groups of subjects.

### 2.7. Evaluation of the Relationship between the Clinical Duration and SRR

Because our cohort has been well documented regarding numerous clinical parameters, we also examined the relationship between the quantified [^99m^Tc]-TRODAT uptake and the clinical duration of PD. A limited number of reports have previously attempted to measure the disease progression rate, as well as the estimated preclinical duration with PET [36, 37] and SPECT [2, 38], in relatively small populations (*n* < 100). In this study, all PD subjects were divided into subgroups according to the clinical duration of their PD symptoms at the time of the TRODAT scan. The grouping was performed on a yearly basis. For example, all PD patients who received a TRODAT within one year since the onset of symptoms were grouped into one group. Within this yearly group, the mean and SD were calculated for the SRR. The same operation was repeated for each year up to fifteen years. If a group from a specific year contained fewer than ten subjects, the group was excluded. This grouping by years of clinical duration resulted in twelve groups. The mean SRR for each group was then plotted as a function of the years of clinical duration. We then used a quadratic function to fit the data points and established the prediction model. With this model, the mean SRR within the healthy control group was extrapolated to estimate the preclinical period, which was defined as the number of years in which the dopaminergic system has been degraded without present and observable clinical symptoms.

We have shared our software as an open source software package at https://sites.google.com/site/deanfanglab/. This software package is free for academic research use.

## 3. Results

**Figure 3:**
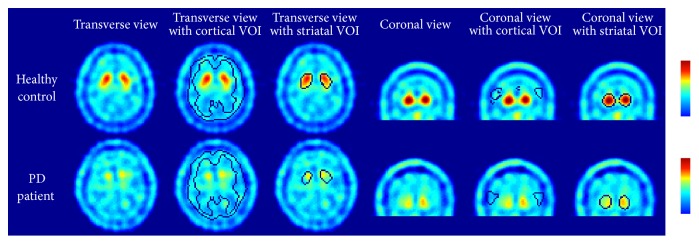
Representative cases of a healthy control and a PD patient with their [^99m^Tc]-TRODAT images aligned with the inversely transformed predefined VOIs for the striatum and the reference region. This figure shows the transverse and coronal views of the original images, the images fused with the reference VOI, and the images fused with the striatal VOI. Through visual interpretation, proper alignment of both VOIs was confirmed and demonstrated.

With the proposed data processing scheme, a fully automated SRR quantification method was implemented in MATLAB based on SPM8. Using the normal template shown in Figure 1, the predetermined striatal and reference VOIs of all subjects were inversely transformed to the images of all subjects. Inverse transformation of the VOIs was visually confirmed to be appropriate for all subjects.  Figure 3 shows two representative subjects, including one healthy control and one PD patient, who had a clinical duration of four years. Both the reference and striatal VOIs were properly transformed in alignment with the original images of both subjects. The means and SD of the SRR for all groups are summarized in Table 2. The SRR dropped from 1.95 ± 0.22 in the nPD group to 1.47 ± 0.19 in the PD group. The subgroups of the PD patients showed a dependency of the SRR decline as a function of the clinical duration. Within the nPD group, the ET patients had a slightly lower SRR mean (1.89) compared with the healthy controls (2.02). The *t*-test identified a significant difference between these two subgroups of nPD subjects (*p* < 0.05).

**Figure 4:**
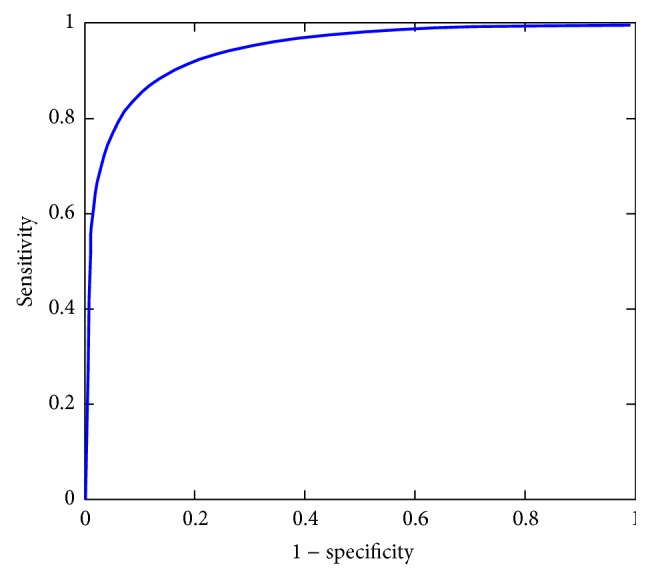
The ROC curve for the differentiation of the PD subjects (*n* = 365) from the nPD subjects (*n* = 61). The area under curve (AUC) was 0.94 for this ROC curve, which indicates an excellent diagnostic performance based on the fully automatic SRR quantification proposed in this report.

The ROC analysis based on the SRR for discriminating nPD from PD subjects is summarized in Table 3.  Figure 4 shows the ROC curve for this test. There was good discriminating power for the SRR between the PD and nPD groups with an AUC of 0.94, a sensitivity of 92%, a specificity of 90%, and an accuracy of 92%. Discriminating healthy controls from PD subjects was easier (AUC = 0.98) compared with the ET subjects (AUC = 0.91). If the PD subjects were further divided based on the clinical duration, the subgroup with two years of clinical duration was the most difficult group to discriminate from the nPD subjects, with an AUC of 0.91 and a sensitivity, specificity, and accuracy of 88%, 90%, and 89%, respectively. The discriminative accuracy increases as the clinical duration increases.  Table 4 shows the diagnosis sensitivity, specificity, and accuracy with the SRR cutoff of 1.73, which was determined by the optimal cutoff for discriminating the PD and nPD groups.

We have compared our method to the conventional method, which applies predefined VOIs on spatially normalized images. For the latter, the AUC of the ROC curve was 0.91, which was worse than the AUC of 0.94 for our method. When the ability to discriminate the PD and nPD groups in the conventional method was compared to our method, the diagnostic accuracy and sensitivity decreased by 10% and 8%, respectively, as shown in Table 5. Comparing between the novel and conventional methods, McNemar's *χ* ^2^ test showed a significant difference in the sensitivity (*p* < 0.05). However, there was not a significant difference for discrimination specificity shown by McNemar's *χ* ^2^ test.

**Figure 5:**
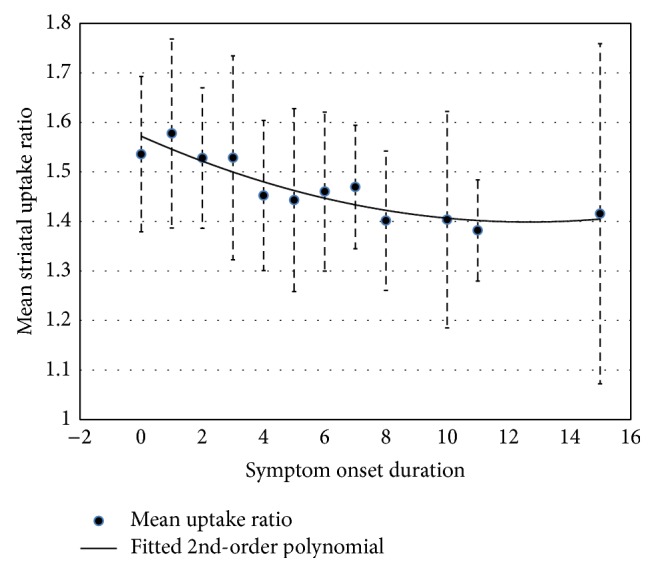
The relationship between the onset duration and the uptake ratio. Each data point represents the SRR mean of that particular year of clinical duration, and the error bars represent the SD. A quadratic function, shown as the solid curve, was used to fit the data points. The *R* ^2^ was 0.8446. Based on this quadratic function and the mean SRR of 2.02 in the healthy controls, a preclinical duration of 11.3 years was estimated with curve extrapolation.

Figure 5 shows the plot of the SRR versus the clinical duration. The means and SD were plotted for each group of patients at individual years for the clinical duration. A quadratic function provided a good fit for the mean SRR for each group as *y* = 0.0011*x* ^2^ − 0.0273*x* + 1.572, where *y* is the mean SRR and *x* is the clinical duration in years with an *R* ^2^ of 0.84. The preclinical duration was estimated with the fitted quadratic function and the mean SRR of the healthy controls. By setting *y* as 2.02 (i.e., the mean SRR of the health controls), we calculated an estimated preclinical duration to be 11.3 years.

## 4. Discussion

In recent years, SPECT-based brain scans have become increasingly common in routine PD diagnosis. Several recent reviews have noted the clinical value of SPECT scans in PD diagnosis and management [9, 15, 39]. However, SPECT scans have their limitations, which primarily result from the poor spatial resolution. Thus, reliable visual interpretation for those scans requires experienced readers, especially for patients in the early stages of the disease [40, 41]. Quantification of the SRR or SUR, as an objective measurement of the tracer uptake, can therefore serve as a useful tool to assist the interpreters in forming a more accurate diagnosis. Compared with other fully automatic methods [14, 20–22], SPM-based methods have become increasingly popular because they are easy to implement and exhibit good discriminative performance. In addition, with its free for academic use policy, SPM has been regarded as the software of choice for neuroimaging studies. The spatial normalization capabilities of SPM matured long ago and have proven their usefulness in numerous studies.

The primary goal of this study was to further improve the diagnostic performance of an SPM-based method that quantifies the SRR, as well as to test how accurately our method discriminates PD patients from healthy controls and ET patients who lack Parkinsonism. The major modification in our method is that we transformed the VOIs from the template domain to the original image domain and subsequently applied these inversely transformed VOIs to the original images to calculate the SRR. In comparison, the conventional method applies the VOIs that are predefined in the template domain to the spatially normalized images. By comparing the ROC analysis results in Table 3 (our method) with Table 5 (conventional method), the advantage of using the inversely transformed VOIs of our method is clear. Higher sensitivity (92.1% versus 82.7%, *p* < 0.05 in McNemar's test) and accuracy (91.8% versus 83.8%) were achieved with our method in the discrimination task that separated the PD from nPD subjects. Using the SRR measured with our fully automatic method, the diagnostic accuracy, sensitivity, and specificity were 92%, 90%, and 92%, respectively, if the clinical duration of PD was not considered and the nPD group included both the healthy controls and the ET subjects. With a good diagnostic performance, our method provides a reliable way to quantify SRR from clinical SPECT brain scans that may further serve as a biomarker for evaluating the disease severity, duration, progression, and therapeutic effects.

If we examine the ROC analysis of the subgroups within the PD and nPD groups, the following observations can be made. First, if we altered the diseased group to include all PD patients, the discrimination was more successful in healthy controls compared with ET subjects. This could be a result of the age difference between the two groups of subjects in this study (52.3 for healthy controls, 72.1 for ET patients). Second, if we attempt to discriminate the nPD group (*n* = 61) from the different subgroups of PD patients, the discrimination was more difficult for the patients in earlier stages. Compared with the nPD group, the difference between Group_≤2_ and Group_≥11_ was 5-6% in accuracy, sensitivity, and specificity. Even for the most challenging group within three years of clinical duration, we still obtained a 90% specificity and approximately 90% accuracy and sensitivity. These findings indicate that the SRR quantified with [^99m^Tc]-TRODAT SPECT images using our method has good diagnostic value even when a patient is in the early stages of the disease.

A large number of patients were included in our study population (PD group, *n* = 365; nPD group, *n* = 66). One advantage of this large population is the validation of diagnostic usefulness of the quantified SRR. The other advantage is that, given the heterogeneous clinical durations in our PD patients, we could divide these patients based on their clinical durations into subgroups and evaluate how this yearly clinical duration correlates with the mean SRR. Our data showed a steady decline in the SRR as a function of the clinical duration with a high correlation (*R* ^2^ = 0.84). Furthermore, at the beginning of the symptom onset, the mean SRR dropped from 2.02 for the healthy controls to 1.5–1.6 for the PD patients. With the quadratic function fitted to our data, we obtained an estimate of 11.3 years as the preclinical duration. This finding adds an additional piece of information to the current perspective of the preclinical duration of PD [42–44]. We speculate that in addition to identifying an estimate of the preclinical duration, image-based SRR quantification may play a significant role in the early determination of dopaminergic neuron degeneration. Early intervention, prior to symptom onset, has high potential for PD treatment [45, 46]. If imaging and its quantification can be further validated for reliability, there is a good chance of identifying molecular degradation several years prior to the appearance of clinical symptoms. As a result, improved therapeutic efficacy may be expected when PD patients are treated by early intervention in the “golden” window.

This study has some limitations in the study design. First, when the disease status of the PD patients was considered, clinical duration was used as the major criteria for grouping the patients. We did not consider the parameters for clinical severity, such as the UPDRS and H&Y scores of these patients. Second, only one scan was used in the data analysis for each patient. A longitudinal evaluation of the SRR regression in serial follow-up scans is a potential future topic for extending this work. Finally, we only used SPECT images for the spatial normalization in this study. Although these spatial normalizations provide satisfactory results, more precise spatial normalization can be expected if anatomic information from CT or MR images is incorporated in the future.

## 5. Conclusion

We have developed a fully automated method, based on SPM spatial normalization, to quantify the striatal tracer uptake for [^99m^Tc]-TRODAT SPECT studies. With a large cohort of more than four hundred subjects, this method showed excellent diagnostic performance with 92% accuracy, 92% sensitivity, and 90% specificity when discriminating the PD subjects from the healthy controls and the ET subjects. A steady degradation of striatal uptake was observed as a function of the clinical duration after an estimated preclinical duration of eleven years prior to PD symptom onset. This method can assist future routine [^99m^Tc]-TRODAT studies, as well as other tracers, in the evaluation of the integrity of dopamine transporters and in the diagnosis of PD patients.

## Figures and Tables

**Table 1:** Demographical summary of the subjects involved in this study. In the Parkinson's disease (PD) group, the subgroups were divided based on clinical duration, which was defined as the number of years between the onset of PD-related symptoms and the TRODAT scan for an individual patient.

	Disease duration, years	*n*	Sex		Age at scan, years				Age of symptom onset, years				Disease duration at scan, years			
			M	F	Mean	SD	Max	Min	Mean	SD	Max	Min	Mean	SD	Max	Min
PD group	**all**	**365**	**222**	**143**	**61.6**	**11.3**	**87**	**28**	**55.9**	**11.7**	**80**	**27**	**5.8**	**5.3**	**33**	**0**
Group_≤2_	≤2	118	70	48	59.8	11.4	78	28	58.6	11.4	77	27	1.2	0.7	2	0
Group_3–5_	3~5	100	63	37	61.3	11.9	83	33	57.4	11.9	80	30	3.8	0.8	5	3
Group_6–10_	6~10	87	50	37	62.1	10.9	87	37	54.6	11.1	80	28	7.5	1.4	10	6
Group_≥11_	≥11	60	39	21	65.0	10.0	85	42	49.5	10.3	70	27	15.5	4.4	33	11
nPD group		**61**	**28**	**33**	**63.0**	**16.3**	**86**	**21**								
Healthy control		28	18	10	52.3	15.7	82	21								
Essential tremor		33	10	23	72.1	10.0	86	49								

**Table 2:** The mean and standard deviation (SD) of the striatal-to-reference ratio (SRR) for the different cohorts.

	Mean	SD
PD group	1.47	0.19
Group_≤2_	1.55	0.17
Group_3–5_	1.48	0.19
Group_6–10_	1.43	0.16
Group_≥11_	1.35	0.17
nPD group	1.95	0.22
Healthy control	2.02	0.20
Essential tremor	1.89	0.23

**Table 3:** The ROC analysis for differentiating the PD patients from the nPD group and its subgroups based on the SRR calculated with our novel method that uses inversely transformed striatal and reference VOIs.

	Versus nPD group					Versus healthy controls					Versus essential tremor subjects				
	Cutoff	Sensitivity	Specificity	Accuracy	AUC	Cutoff	Sensitivity	Specificity	Accuracy	AUC	Cutoff	Sensitivity	Specificity	Accuracy	AUC
PD group	1.73	92.1	90.2	91.8	0.94	1.76	93.2	96.4	93.4	0.98	1.67	87.7	84.8	87.4	0.91
Group_≤2_	1.73	88.1	90.2	88.8	0.91	1.76	90.7	96.4	91.8	0.97	1.73	88.1	81.8	86.8	0.87
Group_3–5_	1.73	89.0	90.2	89.4	0.93	1.75	90.0	96.4	91.4	0.97	1.73	90.0	81.8	88.0	0.90
Group_6–10_	1.66	94.3	91.8	93.2	0.96	1.72	97.7	100.0	98.3	0.99	1.66	94.3	84.8	91.7	0.93
Group_≥11_	1.51	93.3	95.1	94.2	0.97	1.68	96.7	100.0	97.7	0.98	1.47	91.7	97.0	93.5	0.97

**Table 4:** The sensitivity, specificity, and accuracy for discrimination using 1.73 as the cutoff SRR calculated with our method.

	Versus nPD group			Versus healthy controls			Versus essential tremor subjects		
	Sensitivity	Specificity	Accuracy	Sensitivity	Specificity	Accuracy	Sensitivity	Specificity	Accuracy
PD group	92.1	90.2	91.8	92.1	100.0	92.6	92.1	81.8	91.2
Group_≤2_	88.1	90.2	88.8	88.1	100.0	90.4	88.1	81.8	86.8
Group_3–5_	89.0	90.2	89.4	89.0	100.0	91.4	89.0	81.8	87.2
Group_6–10_	97.7	90.2	94.6	97.7	100.0	98.3	97.7	81.8	93.3
Group_≥11_	96.7	90.2	93.4	96.7	100.0	97.7	96.7	81.8	91.4

**Table 5:** The ROC analysis for the discrimination tasks based on the SRR calculated with the conventional method, which applies predetermined VOIs to the spatially normalized images. Better discrimination power was achieved with the inversely transformed VOIs using our new method, as shown in Table 3.

	Versus nPD group					Versus healthy controls					Versus essential tremor subjects				
	Cutoff	Sensitivity	Specificity	Accuracy	AUC	Cutoff	Sensitivity	Specificity	Accuracy	AUC	Cutoff	Sensitivity	Specificity	Accuracy	AUC
PD group	1.65	82.7	90.2	83.8	0.91	1.66	84.1	92.9	84.7	0.93	1.71	92.1	81.8	91.2	0.90
Group_≤2_	1.65	84.7	90.2	86.6	0.92	1.66	87.3	92.9	88.4	0.94	1.71	94.9	81.8	92.1	0.90
Group_3–5_	1.69	87.0	83.6	85.7	0.91	1.69	87.0	85.7	86.7	0.93	1.71	90.0	81.8	88.0	0.90
Group_6–10_	1.65	82.8	90.2	85.8	0.91	1.66	83.9	92.9	86.1	0.92	1.71	90.8	81.8	88.3	0.90
Group_≥11_	1.69	88.3	83.6	86.0	0.92	1.66	83.3	92.9	86.4	0.93	1.71	91.7	81.8	88.2	0.91
